# Screening of Respiratory Tract–Associated Nematodes in Moroccan Dogs: Focus on *Eucoleus boehmi*

**DOI:** 10.1155/vmi/7573637

**Published:** 2026-10-05

**Authors:** Houda Idrissi, Sarah ElHamiani Khatat, Luc Duchateau, Sylvie Daminet, Noureddine Tazi, Issam Fettach, Malika Kachani, Hamid Sahibi

**Affiliations:** ^1^ Department of Pathology and Veterinary Public Health, Hassan II Agronomy and Veterinary Institute, Rabat-Instituts, Madinat Al Irfane P.O. Box 6202, Rabat, Morocco; ^2^ Department of Medicine, Surgery and Reproduction, Hassan II Agronomy and Veterinary Institute, Rabat-Instituts, Madinat Al Irfane P.O. Box 6202, Rabat, Morocco; ^3^ Biometrics Research Center Faculty of Veterinary Medicine Ghent University Salisburylaan 133 Merelbeke 9820 Belgium ugent.be; ^4^ Small Animal Department Faculty of Veterinary Medicine Ghent University Salisburylaan 133 Merelbeke 9820 Belgium ugent.be; ^5^ Veterinary Clinic of the 1st Kennel of the Royal Armed Forces Benslimane 13000 Morocco; ^6^ Military Health Service Es-Semara Military Garrison Royal Armed Forces (FAR) Rabat Morocco; ^7^ College of Veterinary Medicine Western University of Health Sciences 309 E. Second Street Pomona California 91766-1854 USA westernu.edu

**Keywords:** *Angiostrongylus vasorum*, dogs respiratory tract helminths, *Eucoleus boehmi*, seroprevalence

## Abstract

Dogs can harbor a range of respiratory tract helminths which can cause clinical signs varying from asymptomatic to serious diseases. Lately, these parasites, notably *Eucoleus* spp. and *Angiostrongylus vasorum*, received considerable attention because of their spread in previously nonendemic regions, likely facilitated by several factors such as increased animal movements changes in the distribution and urbanization of wild canid reservoirs, along with climatic and environmental changes. This study aimed to assess the presence and dissemination of these helminths by investigating, for the first time in Morocco, the seroprevalence of *A. vasorum* and the fecal occurrence of *Eucoleus* spp. eggs in dogs. Overall, 318 fecal samples collected from dogs across different geographical areas of Morocco were examined using two fecal flotation techniques, centrifugal flotation with 33% zinc sulfate (ZnSO_4_) and simple flotation with Sheather’s sugar solution. Additionally, *A. vasorum* antigen detection was performed on 286 blood samples using an in‐clinic serological test (Angio Detect Test, IDEXX Laboratories). A complete blood count was subsequently performed in dogs that tested positive. Results revealed the occurrence of *Eucoleus boehmi* eggs in three military working dogs (0.94%), with two being asymptomatic and one showing a mucous nasal discharge. All had mild to moderate eosinophilia (1.33–3.18 K/μL). With regard to the serological assays, all dogs tested negative for *A. vasorum* antigens. These preliminary findings provide initial evidence of the geographic spread of *E. boehmi*, suggesting that veterinarians in Morocco should include this infection in the differential diagnosis of dogs displaying upper respiratory tract symptoms. By contrast, despite the absence of dogs seropositive to *A. vasorum*, the disease cannot be excluded until a better understanding of its distribution is established. Hence, further studies involving larger canine populations across different regions and combining multiple diagnostic methods, including advanced molecular techniques, are highly recommended.

## 1. Introduction

Dogs can be infected with a variety of respiratory tract helminths [1–3] that have recently received increasing attention due to their emergence and spread in previously nonendemic regions, including *Eucoleus boehmi*, *E. aerophilus, Angiostrongylus vasorum*, and *Crenosoma vulpis* [2, 4–10]. Several of these helminths, such as *A. vasorum*, have an indirect life cycle involving molluscs as intermediate hosts. By contrast, *Eucoleus* species are generally considered to have a direct life cycle, although earthworms have also been proposed as possible intermediate hosts [1]. Infections with these parasites may lead to various clinical manifestations, ranging from an asymptomatic form to serious conditions [4, 11, 12]. The nonspecificity of the clinical signs associated with respiratory systems often results in misdiagnosis, leaving animals infected and untreated [2]. Moreover, some of these parasites, specifically *E. aerophila*, have zoonotic potential and can cause pulmonary capillariosis in humans, typically presenting nonspecific symptoms that may resemble those of bronchial pneumonia or even lung cancer [5].

In Morocco, research on canine respiratory tract helminths has primarily focused on cardiopulmonary parasites such as *Dirofilaria immitis,* possibly because it is better known and rapid serological tests are available for its diagnosis (SNAP 4DX Plus, IDEXX Laboratories, Inc., Westbrook, ME) [13–15]. Nevertheless, this parasite was not investigated in the present survey. Only recently, a study investigated the occurrence of respiratory tract helminths using the Baermann technique, revealing the occurrence of *A. vasorum* larvae in one dog. However, this case was not confirmed by other diagnostic methods such as serology, which underscored the need for further thorough investigations [15].

Therefore, this study aimed to determine, for the first time in Morocco, the seroprevalence of *A. vasorum* across different categories of dogs and to screen for *Eucoleus* spp. eggs by fecal flotation, thereby improving current knowledge of the epidemiological status of canine respiratory tract helminths [1, 16].

## 2. Materials and Methods

### 2.1. Study Population and Location

A total of 318 fecal samples were collected during two study periods (January 2019–March 2020 and January–April 2022), from various categories of Moroccan dogs, including companion dogs (owned household pets) (*n* = 90), shelter dogs (*n* = 115), and military working dogs (*n* = 107). In addition, 286 blood samples were collected from companion dogs (*n* = 95), shelter dogs (*n* = 109), military working dogs (*n* = 54), and stray/free‐roaming dogs (*n* = 28).

The samples from owned dogs were all collected at the Small Animal Clinic of the Hassan II Agronomy and Veterinary Institute (IAV Hassan II), Rabat, Morocco. The samples from shelter dogs were collected at refuges from the cities of Rabat, Marrakech, and Casablanca. The working dogs were exclusively recruited from a kennel in the city of Benslimane, and the stray/semistray dogs were enrolled from the Rabat‐Salé‐Kénitra region (Ait‐Ichou) (Figure 1).

**FIGURE 1:**
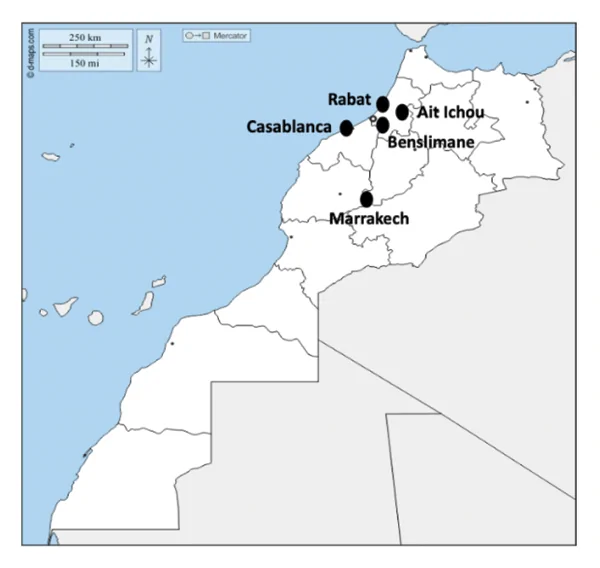
Geographical distribution of the sampled dogs across the study areas in Morocco.

Dogs were recruited using a field‐based convenience sampling strategy. Because no comprehensive sampling frame was available for these heterogeneous dog populations, random sampling was not feasible. Therefore, all dogs meeting the inclusion criteria and accessible during the study period were consecutively enrolled following owner or institutional consent.

### 2.2. Fecal Sample Collection and Analysis

Approximately 30 g of freshly voided feces were collected from each dog and stored at 4°C until processing. For the identification of capillarid eggs, the standard 33% zinc sulfate (ZnSO_4_) centrifugal flotation (specific gravity 1.18) and Sheather’s sugar simple flotation (specific gravity 1.25) techniques were performed within 2 days after sample collection. Eggs were identified and differentiated by microscopic examination according to published keys for the identification of parasites, based on morphological and morphometric criteria, including egg shape and dimensions, bipolar plug morphology, outer shell ornamentation, and embryonic content [16–18].

### 2.3. Blood Sample Collection and Analysis

For each of the 286 dogs, 5 mL of whole blood was collected from the cephalic or jugular vein and divided into a tube with EDTA and a tube without anticoagulant. Serum and plasma were separated by centrifugation and then frozen at −20°C until processing. *A. vasorum* infection was investigated using the IDEXX Angio Detect test, kindly provided by Dr. Adam Gerstein and IDEXX Laboratories, and performed in accordance with the manufacturer’s directions. The test is a rapid in‐clinic immunochromatographic assay developed for the detection of circulating *A. vasorum* antigens in canine serum or plasma. Validation studies have demonstrated high specificity (98.9%–100%) and a sensitivity ranging from 84.6% to 97.1% [19, 20]. No cross‐reactivity was observed with several common canine nematodes, including *C. vulpis* and *Dirofilaria* spp. [19].

### 2.4. Clinical Examination and Hematological Analysis

At the time of sampling, a detailed questionnaire was completed to collect information on each dog’s signalment and medical history, and each animal underwent a thorough physical examination. A complete blood count (CBC) (ProCyte DX, IDEXX Laboratories) was subsequently performed in dogs that tested positive.

## 3. Results

The results revealed the occurrence of *capillarid* eggs in three of the 318 tested samples (0.94%). Eggs had a clear to golden color, had bipolar plugs, measured between 55 and 60 μm in length and 30–35 μm in width, and contained a multicellular embryo that did not completely fill the egg (Figure 2). Careful inspection of the surfaces showed a pitted appearance. These morphological and morphometric features were compatible with those of the upper respiratory tract parasite, *Eucoleus boehmi* [16–18].

**FIGURE 2:**
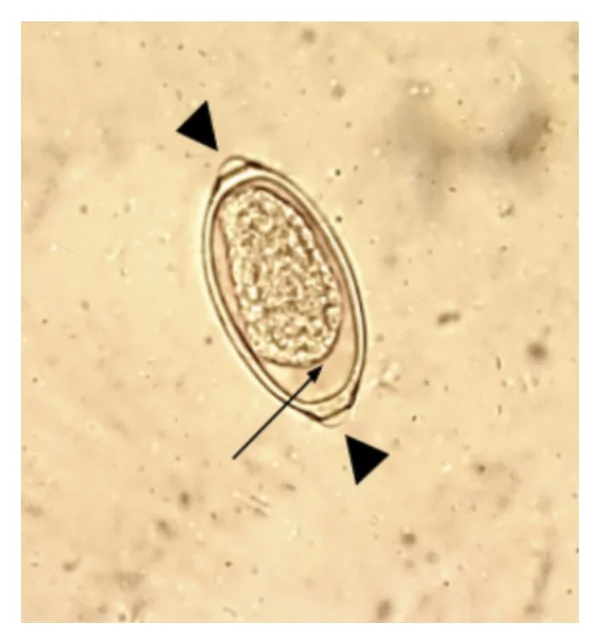
Egg of *Eucoleus boehmi*, clear/gold in color, showing a bipolar plug (arrowheads) and an embryo that does not completely fill the egg (arrow). Flotation, magnification × 100.

The positive fecal samples were collected from three working dogs, aged four, five, and 7 years. They were two females and one male, including two German shepherds and one Belgian Malinois. Two of these dogs were asymptomatic, while one had a mucous nasal discharge. No other abnormalities were detected on physical examination. The CBC showed a moderate eosinophilia in one dog (3.18 K/μL, RF: 0.06–1.23 K/μL) and a mild eosinophilia in the other two (1.58 K/μL and 1.33 K/μL). A mild basophilia (0.15 K/μL, RF: 0.00–0.10 K/μL) and a mild monocytosis were detected in one of these dogs (1.60 K/μL, RF: 0.16–1.12 K/μL) (Table 1).

**TABLE 1:** Signalment, clinical signs, and hematological findings (CBC) of 3 *E. boehmi*‐positive working dogs.

Dogs	1	2	3
Breed	Belgian Malinois	German shepherds	German shepherds
Sex	Female	Female	Male
Age (years)	4	5	7
Abnormal clinical signs	Mucous nasal discharge	—	—
Abnormal CBC results	Eosinophils 1.58 K/μL	Eosinophils 1.33 K/μL	Eosinophils 3.18 K/μL Basophils 0.15 K/μL Monocytes 1.60 K/μL

The serological tests showed that all screened samples were negative to *A. vasorum* antigens.

## 4. Discussion

In Morocco, knowledge regarding canine respiratory tract helminths and their epidemiology is extremely limited [15], which prompted the conduct of this study and highlighted its significance.

The results revealed the occurrence of upper respiratory tract helminths such as *E. boehmi* in the feces of three dogs out of the 318 (0.94%) tested using the flotation technique. To the best of the authors’ knowledge, these are the first reported cases of *E. boehmi* infection in Morocco. In accordance with our findings, infection of dogs with *E. boehmi* is considered to be sporadic throughout the world [21], with only a few cases reported in dogs from various countries, such as in North America (4/230 dogs) [22], Portugal (1/1 dog) [23], Italy (6/270 dogs and 5/639 dogs) [24, 25], South America (2/306 dogs) [26], and Switzerland (3/3 dogs) [27]. It is believed that the reported cases and the prevalence might be underestimated because of potentially misdiagnosed patients. The reasons for this supposition include the variable clinical presentation of this infection and the morphological similarities of *E. boehmi* and other capillarid eggs (*Trichuris vulpis* and *E. aerophilus*), which may lead to misdiagnosis and confusion with other diseases (e.g., mycosis, foreign bodies, and tumors); the cyclical egg shedding requiring multiple fecal examinations; and the location and the small size of the adult parasites [21, 23, 28]. The positive dogs reported in our study were all working dogs, belonging to the same kennel. Indeed, living in overpopulated environments (e.g., kennels, shelters, or breeding facilities) may be a potential risk factor for this infection, because the parasite may concentrate and spread due to its biology and the high resistance of the eggs in soil [21]. Two of these dogs were asymptomatic, while one dog had a mucous nasal discharge. This is in accordance with the literature reporting that *E. boehmi* infections are usually subclinical [16, 21, 23]. However, depending on the parasite load, dogs may display upper respiratory tract signs with varying degrees of severity, such as sneezing, reverse sneezing, and mucopurulent nasal discharges that may be hemorrhagic [1, 21, 23, 28]. The CBC results of the three dogs revealed a mild to moderate eosinophilia that could potentially result from the infection with *E. boehmi* or even may be related to a potential eosinophilic inflammatory infiltrate of the nasal mucosa, which is a common condition noticed during *E. boehmi* infection [23, 29]. These preliminary results suggest a potential spread of *E. boehmi* into new geographical areas and highlight the need for Moroccan veterinarians to include *E. boehmi* infection in the differential diagnosis of dogs displaying upper respiratory tract signs. Thus, diagnostic procedures, such as coprologic and nasal flush examinations, should be performed in suspected dogs to avoid underdiagnosis, which can lead to inappropriate or ineffective treatment [11, 23].

All the blood samples screened for *A. vasorum* antigens tested negative. However, we believe that these results are not sufficient to conclude a real absence of *A. vasorum* infection in Moroccan dogs. Particularly, when considering the suspected case previously reported in Morocco [15], in addition to the proven geographical expansion of *A. vasorum* in previously nonendemic areas and the reported autochthonous cases in several previously free countries [6, 8, 30, 31]. These findings may have been influenced by several factors, such as seasonal and/or sampling biases [32]. In fact, the case previously reported in the country was sampled in Rabat, but according to its owner, the dog was bought from Tangier. This city is in the northwest of Morocco, on the Strait of Gibraltar and its ports, and is one of the main pathways connecting Africa to Europe, in terms of commercial and touristic exchanges. Thus, this region presents a high risk of animal movement, which is considered one of the potential reasons for angiostrongylosis expansion [8, 33]. Thereby, further studies including a larger canine population are highly needed.

Finally, several limitations of this study should be acknowledged. First, the relatively limited sample size and the restricted geographical coverage of the sampling may have affected the representativeness of the study population and the generalizability of the findings. Second, although the use of conventional coproscopic techniques for the detection of *E. boehmi* and serological assays for the detection of *A. vasorum* are well suited for large‐scale epidemiological surveys, relying solely on these diagnostic approaches may have resulted in an underestimation of the true prevalence. The combined use of coprological, serological, and molecular methods could therefore improve overall diagnostic sensitivity and provide a more accurate estimate of the prevalence [34, 35]. Nevertheless, this study provides valuable baseline data on the epidemiology of these helminths in dogs in Morocco and lays the groundwork for future research. Larger‐scale studies including canine populations from different regions of the country, particularly from the Northwest regions, combined with complementary diagnostic approaches (e.g., Baermann of feces sampled over three successive days, serology, and PCR) are highly recommended for a better understanding and appreciation of the epidemiology of these helminths and to inform the development of effective and adapted prevention and control strategies. Furthermore, investigations targeting the potential intermediate hosts of lungworms, particularly *A. vasorum*, would provide valuable insights into its transmission dynamics and epidemiology.

## 5. Conclusion

This study is the first report of *E. boehmi* infection in dogs from Morocco, suggesting a broader geographic distribution than previously reported. These preliminary results are relevant to veterinary practice, suggesting that veterinarians in Morocco should include *E. boehmi* infection in the differential diagnosis of dogs displaying upper respiratory tract signs. Conversely, all sampled dogs were seronegative for circulating *A. vasorum* antigens. However, until the epidemiological situation in Morocco is better understood, veterinarians should consider angiostrongylosis in symptomatic dogs, particularly those unresponsive to treatment.

## Author Contributions

The corresponding author had full access to all of the data in this study and takes complete responsibility for the integrity of the data and the accuracy of the data analysis.

## Funding

This work was carried out with the support of the National Center for Scientific and Technical Research (CNRST) as part of the research excellence grant program (grant number: 2IAV2019, 2019).

## Disclosure

All authors have read and approved the final version of the manuscript. The lead author affirms that this manuscript is an honest, accurate, and transparent account of the study being reported; that no important aspects of the study have been omitted; and that any discrepancies from the study as planned and, if relevant, registered have been explained.

## Ethics Statement

This study was approved by the Ethics Committee of Biomedical Research of the Mohammed V University, Faculty of Medicine and Pharmacy and Faculty of Dental Medicine‐Rabat (no: 37/20).

## Conflicts of Interest

The authors declare no conflicts of interest.

## Data Availability

The data generated and analyzed during this study are available from the corresponding author upon reasonable request.
